# The effect of increasing the supply of skilled health providers on pregnancy and birth outcomes: evidence from the midwives service scheme in Nigeria

**DOI:** 10.1186/s12913-016-1688-8

**Published:** 2016-08-23

**Authors:** Edward Okeke, Peter Glick, Amalavoyal Chari, Isa Sadeeq Abubakar, Emma Pitchforth, Josephine Exley, Usman Bashir, Kun Gu, Obinna Onwujekwe

**Affiliations:** 1 RAND Corporation, Santa Monica, CA USA; 2 University of Sussex, Brighton, UK; 3 Bayero University Kano, Kano, Nigeria; 4 RAND Europe, Cambridge, UK; 5 University of Nigeria Enugu, Enugu, Nigeria

**Keywords:** Health workers, Midwives, Supply, Maternal health, Impact evaluation

## Abstract

**Background:**

Limited availability of skilled health providers in developing countries is thought to be an important barrier to achieving maternal and child health-related MDG goals. Little is known, however, about the extent to which scaling-up supply of health providers will lead to improved pregnancy and birth outcomes. We study the effects of the Midwives Service Scheme (MSS), a public sector program in Nigeria that increased the supply of skilled midwives in rural communities on pregnancy and birth outcomes.

**Methods:**

We surveyed 7,104 women with a birth within the preceding five years across 12 states in Nigeria and compared changes in birth outcomes in MSS communities to changes in non-MSS communities over the same period.

**Results:**

The main measured effect of the scheme was a 7.3-percentage point increase in antenatal care use in program clinics and a 5-percentage point increase in overall use of antenatal care, both within the first year of the program. We found no statistically significant effect of the scheme on skilled birth attendance or on maternal delivery complications.

**Conclusion:**

This study highlights the complexity of improving maternal and child health outcomes in developing countries, and shows that scaling up supply of midwives may not be sufficient on its own.

## Background

One of the major global health challenges of the 21st century is reducing the approximately 3 million newborn deaths, 7 million under-five deaths, and 300,000 maternal deaths that occur globally each year. This health burden is not uniformly distributed: most deaths occur in the poorest regions of the world—87 % of maternal deaths and about 65 % of neonatal deaths, for example, occur in sub-Saharan Africa and South Asia [1]. Many of these deaths are believed to be preventable: it has been estimated, for example, that up to a third of maternal deaths, and half of newborn deaths can be prevented by increasing coverage rates for skilled attendance at delivery [2–4].

In many of the countries lagging behind Millennium Development Goal (MDG)-related targets, poor access to skilled health providers, particularly in rural areas, is regarded as one of the main challenges to increasing rates of skilled birth attendance and improving outcomes for children and mothers [5]. Two recent studies have brought this issue into sharp focus by estimating the effect of scaling up access to midwives on maternal and infant health [6, 7]. Given limited empirical evidence regarding the effects of scaling up access to midwives, both studies rely on model-based projections.

In this paper, we present empirical findings from an evaluation of a large-scale program in Nigeria that sought to increase access to midwives in rural communities. This program, known as the Midwives Service Scheme, deployed thousands of midwives to primary health facilities across Nigeria to increase access to skilled care. The stated goal of the program was to double the proportion of births attended by skilled attendants by December 2015. In 2014 we carried out a mixed-methods evaluation to study the impact of the program on use of antenatal care during pregnancy and on skilled birth attendance five years after implementation of the scheme. We also examine effects on maternal delivery complications. This study provides timely evidence to policy makers in developing countries looking to increase coverage of skilled birth attendance and improve maternal and child health outcomes.

## Methods

### The Nigerian midwives service scheme

Every year, more than 50,000 Nigerian women die from pregnancy-related complications. The chance of a woman dying during pregnancy and childbirth in Nigeria is approximately 1 in 30 compared to about 1 in 2,400 in developed countries [8]. Infants also experience poor health outcomes with an estimated 250,000 newborn deaths annually. These high rates of mortality have been attributed in part to low utilization of pregnancy and delivery care: only 39 % of births, for example, are attended by a skilled health provider. In many health facilities across the country, there is a shortage of skilled providers (doctors, nurses and midwives). A survey of primary health facilities in rural communities found that up to half did not have a single midwife [9].

The Midwives Service Scheme (MSS) was introduced in December 2009 to address these challenges. The key feature of the MSS was the recruitment and deployment of unemployed, retired, and newly graduating midwives to government primary health facilities in rural and underserved communities. Participating midwives were recruited through a national recruitment exercise. The scheme was funded by debt relief funds under a 2009 Appropriations Act and was designed to be a collaborative effort between all three tiers of government. The federal government recruited and deployed the midwives, paid them a monthly salary (N30,000 or approximately 150 USD) and supplied clinics with midwifery kits (these kits contain essential items needed for deliveries such as instruments, sutures, gloves, and cord clamps), basic equipment such as blood pressure apparatus and weighing scales, some essential drugs, and facility/community registers for record keeping; state governments paid additional allowances to midwives (N20,000 per month) and provided monitoring and supervision; local governments paid a supplementary allowance of N10,000 and provided free accommodation for the midwives in the local community.

The first phase of the scheme—the subject of our evaluation—rolled out nationally in 652 primary health care facilities in 2009. The distribution of MSS facilities was determined largely by geographic location. States in the northeast and northwest zones (classified as ‘very high’ maternal mortality zones) were assigned 24 facilities each, states in the north-central and south-south (classified as ‘high’ mortality zones) were assigned 16 facilities each, and states in the southwest and southeast (classified as ‘moderate’ mortality zones) were assigned 12 facilities each (see Table 1). Each MSS facility was linked to a general hospital that would serve as a referral hospital. Nearly 2,500 midwives in total were deployed in the first phase of the scheme.

**Table 1:** Distribution of health facilities

Region	Number of states/region	Number of clinics/state
North-East	6	24
North-West	7	24
North-Central	7	16
South-South	6	16
South-East	5	12
South-West	6	12

### Study design

**Fig. 1:**
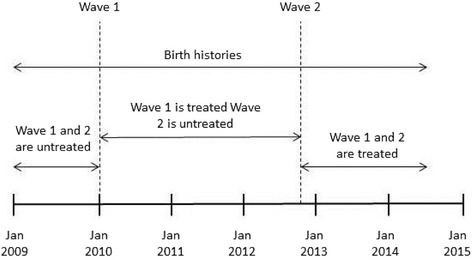
Graphical illustration of research design

To identify the effects of the MSS we compared changes in pregnancy and birth outcomes in MSS (intervention) communities to changes in non-MSS (comparison) communities in the same states over the same period i.e., a difference-in-difference design. The comparison group consisted of similar communities (as the intervention communities) that, however, were not enrolled in (an updated version of) the program until three years later; we refer to this as Wave 2. Thus we exploit the fact that there was a window of time within which one group of communities was exposed (Wave 1) but the other was not yet exposed (Wave 2). Both sets of health facilities were selected using the same criteria. Data on pregnancy and birth outcomes were collected retrospectively through a household survey in both sets of communities targeting women who had a recent pregnancy (see next section). A graphical illustration of our study design is provided in Fig. 1.

Two hundred eight MSS communities were randomly selected to participate in the study. To draw the study sample we began by randomly selecting two states in each geopolitical zone (making 12 states in total), and then enrolling all MSS health facilities in the state into the study. We randomly selected an equivalent number of Wave 2 facilities in each state to serve as a comparison group. It is important to clarify that prior to rollout, participating communities did not have any advance knowledge that they would participate in the scheme. We carried out qualitative interviews and focus groups in three purposively selected states to shed light on program implementation and to provide additional context for the evaluation findings. These results are presented in a companion paper [10].

### Data collection

Institutional Review Boards at RAND, Bayero University Kano, and University of Nigeria Enugu provided ethical review and approval for the study. Data collection took place between June 2014 and January 2015. Since a comprehensive listing of households in each community was unavailable, we randomly generated Global Positioning System (GPS) coordinates within each community using a GPS-enabled tablet and special software and selected the dwelling nearest this point for interview. Twenty households were randomly selected in each community, with the criterion for inclusion that they contained a woman who was pregnant at least once between January 2009 and the date of interview. If there was no eligible household within the dwelling, the interviewer visited the dwelling on either side until one was found. If there were multiple eligible households within the dwelling, one was randomly chosen for interview. All eligible women within each selected household were interviewed. To obtain consent, interviewers read out a statement to study respondents describing the study and any associated risks/benefits of participation. Interviewers checked a box on the consent screen of the tablet to indicate that verbal consent had been provided. In total we interviewed 7,104 women in 368 communities (not all communities selected could be surveyed because of logistical constraints).

The survey instrument included a household module that collected information including dwelling characteristics, source of drinking water, toilet facilities and possession of various assets (these were aggregated into a wealth index using principal component analysis); and an individual module administered to all eligible women. The individual module collected retrospective information about each birth since January 2009 including use of antenatal and postnatal care, place of delivery, and pregnancy and delivery complications such as haemorrhage, fever and convulsions. Respondents were asked about complications during delivery (intrapartum complications) and within six weeks after delivery (postpartum complications). We also carried out a clinic survey in each study clinic (both MSS and non-MSS clinics). Interviewers collected data on clinic characteristics including staffing and availability of supplies from the officer-in-charge (or another knowledgeable individual if the officer-in-charge was unavailable). All interviews were administered face-to-face using Android tablets.

### key variables

Our primary outcomes are antenatal care and skilled birth attendance. We consider the following measures for antenatal care: (1) an indicator for any antenatal care use, (2) an indicator for whether a mother received at least 4 antenatal visits (per World Health Organization recommendations), and (3) an indicator for antenatal care obtained in the study clinic. For skilled birth attendance our measures are: (1) an indicator for whether a birth took place in the study clinic, (2) an indicator for whether the birth took place in any health facility (i.e., was attended), and (3) an indicator for whether a birth was attended by a doctor, nurse or midwife independent of birth location. For both outcomes we look separately at uptake in the study clinic and overall uptake. This allows us to measure any substitution effects i.e., women switching from other sources of formal care to the MSS clinic. Even though the goal of the program was to increase uptake among prior non-users, switching from other sources of formal care to the MSS clinic is potentially beneficial if the MSS clinic is closer, thus reducing time and travel costs, and/or if the care offered by MSS midwives is of higher quality relative to existing alternatives.

Our secondary outcomes are maternal birth complications. We look at the probability that a mother experienced at least one of several complications including severe bleeding, convulsions, retained placenta, prolonged labor, loss of consciousness or high fever, either during the delivery (intrapartum complications) or within six weeks of delivery (postpartum complications).

### Empirical strategy

To identify the impact of the MSS, we estimate difference-in-difference (DID) models that examine the relative change in outcomes in MSS (intervention) relative to non-MSS (comparison) areas. The basic econometric specification is the following:

1 $$ {y}_{ijt}=\alpha +{\beta}_1 Treate{d}_j+{\beta}_2Pos{t}_t+{\beta}_3 Treate{d}_j*Pos{t}_t+{\eta}_j+{e}_{ijt} $$

*y*_*ijt*_ denotes the outcome of interest for birth *i* in community *j* in month *t* (starting in January 2009); *Treated*_*jt*_ is an indicator that takes the value 1 if the study clinic in community *j* is a Wave 1 (MSS) clinic; *Post*_*t*_ is a binary indicator that takes the value 1 after the MSS is introduced; *η*_*j*_ is a community fixed effect; and *e*_*ijt*_ is an unobserved error term. In this specification our interest centers on the coefficient *β*_3_*,* which measures the differential change in the outcome in intervention communities relative to comparison communities. We include controls for gender of the baby, an indicator for a multiple birth, marital status, mother’s education and religion, the mother’s age at the time of the birth, an indicator equal to one if the woman reported at least one complication during pregnancy, and household wealth (quintiles of an asset-based wealth index derived using principal component analysis). Standard errors are clustered at the level of the community given correlation in the outcomes within this level.

The main assumption required for identifying causal effects in the DID model is that the evolution of outcomes in intervention areas would have followed the same pattern as in comparison areas in the absence of the treatment (this is known as the parallel trends assumption). While this counterfactual cannot be known, we can test whether this assumption holds for each of the outcome variables of interest prior to the introduction of the program. Finding that the trends are the same in intervention and control areas before the program adds to confidence in the assumption that (non-program) trends are the same following program introduction as well. We estimate the following regression specification:

2 $$ {y}_{ijt}=\alpha +\beta Treate{d}_j+\gamma t+\delta Treate{d}_j*t+{\eta}_j+{e}_{ijt} $$

where the regression sample is restricted to baseline births (those that occurred before introduction of the MSS), *t* denotes monthly pre-trends, and where interest centers on the interaction coefficient *δ* (the parallel trends assumption implies that *δ = 0*).

## Results

All data were analyzed using Stata 12 software. The survey sample consists of 9,475 births born to 7,104 women over the period 2009–2014, of which 4,746 (50.33 %) occurred in MSS (intervention) areas. We exclude births occurring after women in the comparison group became exposed to the intervention, leaving us with 5,295 births taking place between January 2009 and May 2012. Table 2 summarizes the variables used in the analysis at baseline, and tests for balance across intervention and comparison areas. Even though the DID identification strategy does not require it (as noted earlier, the key assumption involves equivalence of trends not levels), it is reassuring to note that the outcome variables as well as the covariates are relatively well balanced at baseline. In Table 3, we test the parallel trends assumption for each of the outcome variables of interest, using the specification shown in (2). As Table 3 shows, the null hypothesis cannot be rejected for any of the outcome variables, which lends credence to the empirical strategy.

**Table 2:** Baseline characteristics and balance

	Control		Intervention		
	Mean	Std dev	Mean	Std dev	*p-value*
Any antenatal care	0.801	0.400	0.834	0.372	0.281
4+ antenatal visits	0.504	0.500	0.506	0.500	0.953
Antenatal care in study clinic	0.539	0.499	0.622	0.485	0.054
Intra-partum complications	0.064	0.301	0.048	0.240	0.349
Post-partum complications	0.044	0.233	0.042	0.228	0.870
Institutional delivery	0.541	0.499	0.570	0.496	0.496
Delivered in study clinic	0.344	0.475	0.420	0.494	0.058
Skilled birth attendance	0.533	0.499	0.540	0.499	0.876
Breastfed for 6 months	0.417	0.493	0.376	0.485	0.342
Married	0.869	0.338	0.876	0.329	0.761
Age of mother	31.79	40.70	29.31	6.598	0.172
Illiterate	0.542	0.499	0.564	0.496	0.649
Muslim	0.591	0.492	0.574	0.495	0.773
Wealth index	2.934	1.468	2.936	1.431	0.989
Male child	0.512	0.500	0.532	0.499	0.503
Multiple birth	0.029	0.168	0.024	0.153	0.724
Low risk (no problems during pregnancy)	0.828	0.378	0.833	0.374	0.870

*Note*: *p-*values correspond to tests for differences in means, and allow for observations to be correlated within communities

**Table 3:** Testing for differential pre-trends in outcome variables

	Number	δ (95 % CI)		*p-*value
Antenatal care in study clinic	1094	0.002	(−0.020, 0.025)	0.854
Any antenatal care	1091	0.009	(−0.011, 0.029)	0.371
4+ antenatal visits	1094	0.003	(−0.021, 0.028)	0.783
Delivery in study clinic	1094	0.004	(−0.019, 0.026)	0.761
Institutional delivery	1094	0.005	(−0.018, 0.028)	0.684
Skilled birth attendance	1094	−0.001	(−0.023, 0.021)	0.916
Intra-partum complications	1094	0.014	(−0.004, 0.032)	0.126
Post-partum complications	1094	0.008	(−0.005, 0.021)	0.213

δ denotes the differential linear trend in intervention communities relative to comparison communities over the period prior to the start of the MSS. The regression specification controls for community fixed effects. Standard errors in parentheses are clustered at the community level

Table 4 presents the DID results for each of the measures of antenatal care. For each outcome, we report the average effect of the MSS [this is the coefficient on the interaction of *Treated* and *Post* from specification (1)]. We then disaggregate this average effect into effects in each year of the program to examine whether the effects of the scheme vary over time. The impact of the scheme might increase over time if it takes time for information about midwife availability in the clinic to spread through the community or if it takes time to gain the trust of the community. Conversely it might decrease if over time the availability of midwives in the clinic becomes compromised. We present effects for Year 1, Year 2, and Year 3 of the program after which the comparison group becomes exposed.

**Table 4:** Estimated effects of MSS on antenatal care utilization

	Number	β (95 % CI)	*p-*value	%D
Antenatal care in study clinic				
Average effect	5295	0.023 (−0.038,0.084)	0.459	0.037
Year 1	5295	0.073** (0.003,0.142)	0.042	0.117
Year 2	5295	−0.007 (−0.078, 0.063)	0.838	−0.011
Year 3	5295	0.003 (−0.076, 0.082)	0.940	0.005
Any antenatal care				
Average effect	5287	0.018 (−0.025, 0.061)	0.416	0.022
Year 1	5287	0.050* (−0.002,0.102)	0.059	0.060
Year 2	5287	−0.005 (−0.056, 0.045)	0.835	−0.006
Year 3	5287	0.009 (−0.047, 0.064)	0.763	0.011
4+ antenatal visits				
Average effect	5295	−0.006 (−0.075, 0.062)	0.857	−0.012
Year 1	5295	0.028 (−0.047, 0.104)	0.463	0.055
Year 2	5295	−0.036 (−0.119, 0.048)	0.402	−0.071
Year 3	5295	−0.011 (−0.101, 0.080)	0.815	−0.022

β denotes the estimated effect of the MSS program, and is obtained using a differences-in-differences specification that includes month of birth and community fixed effects and controls for maternal and child characteristics. %D expresses the estimated program effect as a percentage of the baseline average of the outcome variable. Standard errors in parentheses are clustered at the community level

**p < 0.05, *p < 0.1

The average effect in Table 4 indicates a small positive impact of the program on antenatal care although this result is not statistically significant. We see, however, that this average effect obscures significant variation over time. Row 2 indicates that the rate of antenatal care usage at MSS clinics increased by about 7.3 percentage points (CI: 0.3 to 14.2 %) in the first year of the program (starting from a baseline rate of 62.2 %), but thereafter we are not able to find any program effect. There is a less precisely estimated 5-percentage point increase (CI: −0.2 to 10.2 %) in the overall rate of antenatal care use, but little evidence of an increase in the number of visits as measured by the rate of four or more antenatal visits.

**Fig. 2:**
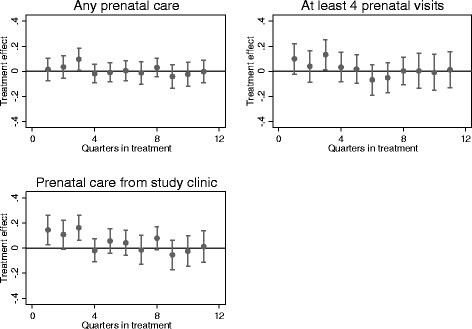
Program effects over time: Antenatal care

In Fig. 2, we plot the coefficients (and associated 95 % confidence intervals) from a more refined specification that allows program effects to vary by quarter. We find that antenatal care usage at the study clinic rose by more than 10%age points in the first three quarters following introduction of MSS, but thereafter dropped off. The graphs also show that overall use of antenatal care (not restricting to care in the study clinic) increased on both the extensive (i.e. rate of any antenatal care) and intensive margins (rate of four or more antenatal visits) during this period, but thereafter declined. From this we conclude that the MSS had a significant, but somewhat short-lived, impact on antenatal care use.

**Fig. 3:**
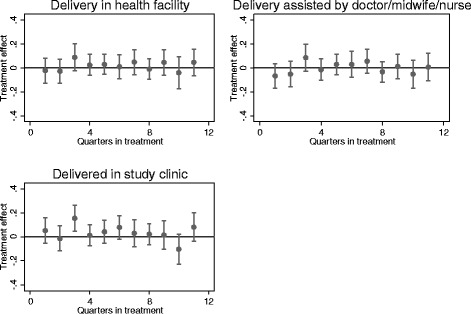
Program effects over time: Delivery

Table 5 presents the DID results for each of the measures of skilled birth attendance. Again we report average effects and effects over time. Overall, the MSS appears to have had little impact on delivery in the study clinic (Estimate: −0.7 %; CI: −7.0 to 5.5 %), institutional delivery (Estimate: −1.6 %; CI: −7.4 to 4.1 %), or skilled birth attendance (Estimate: −0.16 %; CI: −5.6 to 5.5 %). The confidence intervals are tight enough that we can rule out economically significant effects on any of the outcomes. This conclusion is not significantly modified if we decompose program effects by year or quarter (see Fig. 3).

**Table 5:** Estimated effects of MSS on institutional delivery and skilled birth attendance

	Number	β (95 % CI)	*p-*value	%D
Delivery in study clinic				
Average effect	5295	−0.007 (−0.070, 0.055)	0.815	−0.017
Year 1	5295	−0.008 (−0.081, 0.065)	0.833	−0.019
Year 2	5295	0.005 (−0.067, 0.078)	0.883	0.012
Year 3	5295	−0.022 (−0.099, 0.055)	0.567	−0.052
Institutional delivery				
Average effect	5295	−0.016 (−0.074, 0.041)	0.574	−0.028
Year 1	5295	−0.035 (−0.104, 0.035)	0.331	−0.061
Year 2	5295	0.000 (−0.066, 0.066)	0.991	0.000
Year 3	5295	−0.016 (−0.085, 0.053)	0.657	−0.028
Skilled birth attendance				
Average effect	5295	−0.000 (−0.056, 0.055)	0.987	0.000
Year 1	5295	−0.014 (−0.081, 0.054)	0.686	−0.026
Year 2	5295	0.025 (−0.041, 0.090)	0.458	0.046
Year 3	5295	−0.015 (−0.084, 0.053)	0.665	−0.028

β denotes the estimated effect of the MSS program, and is obtained using a differences-in-differences specification that includes month of birth and community fixed effects and controls for maternal and child characteristics. %D expresses the estimated program effect as a percentage of the baseline average of the outcome variable. Standard errors in parentheses are clustered at the community level

In Table 6 we examine the impact of the MSS on our secondary outcome, maternal complications. Not surprisingly given the lack of major impacts on antenatal care or birth attendance, we find no evidence that the MSS reduced the incidence of intrapartum complications (Estimate: 1.2 %; CI: −2.8 to 5.1 %) or postpartum complications (Estimate: 0.2 %; CI: −3.3 to 3.8 %).

**Table 6:** Estimated effects of MSS on maternal complications

	Number	β (95 % CI)	*p-*value	%D
Intra-partum complications				
Average effect	5295	0.012 (−0.028, 0.051)	0.556	0.250
Year 1	5295	0.003 (−0.044, 0.045)	0.912	0.063
Year 2	5295	0.014 (−0.035, 0.063)	0.577	0.292
Year 3	5295	0.020 (−0.033, 0.073)	0.466	0.417
Post-partum complications				
Average effect	5295	0.002 (−0.033, 0.038)	0.891	0.048
Year 1	5295	−0.014 (−0.053, 0.024)	0.469	−0.333
Year 2	5295	0.010 (−0.038, 0.052)	0.660	0.238
Year 3	5295	0.013 (−0.037, 0.063)	0.612	0.310

β denotes the estimated effect of the MSS program, and is obtained using a differences-in-differences specification that includes month of birth and community fixed effects and controls for maternal and child characteristics. %D expresses the estimated program effect as a percentage of the baseline average of the outcome variable. Standard errors in parentheses are clustered at the community level

## Discussion

The results in the previous section show that the MSS had smaller than anticipated effects (relative to the ambitious program goals). The main measured effect of the program is that it increased the use of antenatal care, with gains concentrated in the first year of the program. The pattern of results indicate that the MSS did not simply result in women changing where they attended antenatal care, as would have been the case if antenatal care use at the study clinic increased without an increase in the overall rate of antenatal care use. Given that the overall rate of antenatal care use increased, albeit by a smaller fraction than the observed increase in the MSS clinics, we conclude that the MSS was successful in inducing some women into antenatal care who would not have used care otherwise. However, we find no evidence of an increase in institutional deliveries or skilled birth attendance, even though this was the primary objective of the scheme. Our confidence intervals allow us to rule out effects larger than about 5.5 percentage points (or about 10 percent of the baseline mean).

The fact that there were some positive impacts at the beginning of the scheme that appeared to erode over time may indicate that the effectiveness of the program was compromised by operational challenges that emerged over time. An in-depth look at the implementation of the scheme [10], indicates that problems such as irregular payment of midwife salaries, and inadequate provision of accommodation, affected availability of midwives in the clinics and contributed to long-run difficulties in retaining midwives in the scheme. However this does not explain why there was no effect on skilled birth attendance, even in the first year of the program. Our data suggest that part of the reason for this is that other dimensions of service quality did not improve, which deterred uptake [10]. For example, data from the clinic survey suggest that clinic infrastructure in many cases remained poor, as did availability of drugs and supplies. Only 44 % of facilities visited received a rating of “good” by project staff regarding the physical condition of the building [options were poor (requires major rehabilitation), fair (requires minor rehabilitation), and good (requires no rehabilitation)]. Lack of electricity and water were also problems: 35 % of MSS clinics in the clinic survey reported having no electricity. Availability of essential medicines and basic equipment was poor. We assigned clinics a score of one for each piece of equipment that was functional, zero if not. The median score was 13 out of 22. With regards to essential medicines, on average, clinics had only about half of these medicines in stock, and 21 % of clinics did not have availability of any of the drugs.

We also find some suggestive evidence that that demand-side barriers such as low perceived need for services and lack of transportation to clinics continued to play an important role (the revised iteration of the scheme sought to address this constraint by including a conditional cash transfer to households to encourage utilization) [11]. For example, in our survey we asked women who gave birth at home why they did not opt for a facility birth, and for 70 % of births that did not take place in a health facility, the mother reported that the reason was because it was “not necessary”. In contrast only 4 % and <1 % of the time did the mother give “facility not open” and “no female provider” as the reason why. This suggests that women did not consider a facility birth to be a high priority.

This study contributes to a growing literature evaluating the effects of policies and programs designed to increase use of maternal and child health services in developing countries. Much of this literature has focused on demand-side initiatives such as conditional cash transfers [12, 13], transportation subsidies [14], and voucher schemes [15–17]; supply-side studies are considerably less common. [18] Limited availability of skilled providers, particularly in rural areas, is thought to be an important constraint but there is little empirical research evaluating the effects of scaling up access to providers. Frankenberg et al. [19], one of the few examples, find weak evidence that the village midwife programme in Indonesia increased use of antenatal care and the likelihood of delivering with the assistance of a medically trained provider. Fauveau et al. [20] study a similar midwife program in Bangladesh and find that many home births were still not attended by midwives. The present study is largely consistent with this work in finding small effects of the Midwifery Scheme in Nigeria and highlights the challenge of improving maternal and child outcomes in developing countries.

This study has several limitations. First, as the evaluation was carried out *ex-post* it presented a number of challenges. For example, though some data were collected at baseline from MSS communities, there was no comparison group at baseline. As such, we do not have a baseline and follow-up in the classical sense; our birth panel is constructed retrospectively. Our design relies on similar trends in intervention and comparison communities, and we attempt to validate this assumption by examining trends in the pre-period. We are not able to reject the null of similarity, but we add the caveat that we have about 11 months of pre-data. A longer pre-period would have been desirable, allowing for a stronger test, but would have meant extending the recall period beyond five years (we followed the widely used Demographic and Health Surveys in keeping the recall period to the last five years). If the underlying assumptions are met, then this strategy provides robust estimates of the effect of the program, but readers should interpret these estimates with this caveat in mind. Second, our approach relies on retrospective information collected from women via a survey, raising the potential problem of accuracy of recall. This is unlikely to be a major concern for our primary outcomes, antenatal care and place of birth/skilled birth attendance, but may be a concern for delivery complications [21, 22]. If the measurement error in the dependent variable is classical (to the extent that it is present) then the estimate of program effects is unbiased (although the standard errors would be larger), but if it is correlated with the explanatory variables then the estimates may be biased [23]. Given this, the results for maternal complications (our secondary outcome) should be interpreted cautiously. However, we note that in developing country settings where a large fraction of births occur outside health institutions, survey data are usually the only option as birth registration data, where available, are notoriously incomplete [24].

## Conclusions

Poor access to skilled health providers, particularly in rural areas, is regarded as one of the main challenges to increasing rates of skilled birth attendance in poor countries. Given the dearth of empirical evidence, researchers have attempted to use models to project the effects of scale-up. Homer et al. [6], for example, estimate that a modest increase in coverage of midwifery could potentially avert 30 % of maternal deaths and half of neonatal deaths. The results in this paper reinforce the need for caution in generalizing from these estimates. As this evaluation shows, scaling up the supply of midwives in the real world is a complex undertaking and may not translate into the desired outcomes. One of the lessons of this evaluation is that increasing the supply of midwives, by itself, may not be a sufficient condition for increasing skilled birth attendance. Other interventions targeting other aspects of service quality such as improving clinic infrastructure and ensuring that clinics have adequate equipment and supplies, strengthening incentives for health providers to deliver high quality care, and tackling demand-side barriers will likely prove necessary.

## Abbreviations

DID: Difference-in-difference
GPS: Global positioning system
MDG: Millennium development goals
MSS: Midwives service scheme
NPHCDA: National primary health care development agency
